# Effects of an mHealth App (Kencom) With Integrated Functions for Healthy Lifestyles on Physical Activity Levels and Cardiovascular Risk Biomarkers: Observational Study of 12,602 Users

**DOI:** 10.2196/21622

**Published:** 2021-04-26

**Authors:** Rikuta Hamaya, Hiroshi Fukuda, Masaki Takebayashi, Masaki Mori, Ryuji Matsushima, Ken Nakano, Kuniaki Miyake, Yoshiaki Tani, Hirohide Yokokawa

**Affiliations:** 1 Division of Preventive Medicine Department of Medicine Brigham and Women’s Hospital and Harvard Medical School Boston, MA United States; 2 Department of Epidemiology Harvard University Boston, MA United States; 3 Department of General Medicine, School of Medicine Juntendo University Tokyo Japan; 4 Department of Advanced Preventive Medicine and Health Literacy, Graduate School of Medicine Juntendo University Tokyo Japan; 5 Graduate School of Health Science Aomori University of Health and Welfare Aomori Japan; 6 DeSC Healthcare Inc. Tokyo Japan

**Keywords:** mHealth, app, cardiovascular disease, physical activity, smartphone, mobile phone

## Abstract

**Background:**

Mobile health (mHealth) apps are considered to be potentially powerful tools for improving lifestyles and preventing cardiovascular disease (CVD), although only few have undergone large, well-designed epidemiological research. “kencom” is a novel mHealth app with integrated functions for healthy lifestyles such as monitoring daily health/step data, providing tailored health information, or facilitating physical activity through group-based game events. The app is linked to large-scale Japanese insurance claims databases and annual health check-up databases, thus comprising a large longitudinal cohort.

**Objective:**

We aimed to assess the effects of kencom on physical activity levels and CVD risk factors such as obesity, hypertension, dyslipidemia, and diabetes mellitus in a large population in Japan.

**Methods:**

Daily step count, annual health check-up data, and insurance claim data of the kencom users were integrated within the kencom system. Step analysis was conducted by comparing the 1-year average daily step count before and after kencom registration. In the CVD risk analysis, changes in CVD biomarkers following kencom registration were evaluated among the users grouped into the quintile according to their change in step count.

**Results:**

A total of 12,602 kencom users were included for the step analysis and 5473 for the CVD risk analysis. The participants were generally healthy and their mean age was 44.1 (SD 10.2) years. The daily step count significantly increased following kencom registration by a mean of 510 steps/day (*P*<.001). In particular, participation in “Arukatsu” events held twice a year within the app was associated with a remarkable increase in step counts. In the CVD risk analysis, the users of the highest quintile in daily step change had, compared with those of the lowest quartile, a significant reduction in weight (–0.92 kg, *P*<.001), low-density lipoprotein cholesterol (–2.78 mg/dL, *P*=.004), hemoglobin A_1c_ (HbA_1c_; –0.04%, *P*=.004), and increase in high-density lipoprotein cholesterol (+1.91 mg/dL, *P*<.001) after adjustment of confounders.

**Conclusions:**

The framework of kencom successfully integrated the Japanese health data from multiple data sources to generate a large, longitudinal data set. The use of the kencom app was significantly associated with enhanced physical activity, which might lead to weight loss and improvement in lipid profile.

## Introduction

Reducing the burden of cardiovascular disease (CVD) is an urgent public health issue. The major aspect of primordial prevention is self-care behavior for the prevention of CVD, such as healthy diet and exercise [1]. Long-term success in lifestyle modification is multifactorial and needs cost-effective, innovative approaches. With the widespread use of smartphones in the last decade, numerous mobile health (mHealth) apps have been developed and considered as potentially powerful tools for this purpose because of their convenience and practicality [2]. The core functions of mHealth apps are self-monitoring, providing tailored information or feedback, and exercise goal setting/reviewing [3,4]. Several apps have undergone scientific evaluation for health effects, and meta-analyses have implicated the favorable effects of mHealth apps on physical activity [5], behavioral change [6], and multiple CVD risk factors [3]. However, previous studies generally targeted small populations and may also have serious selection bias, warranting the need for well-designed epidemiological research [3].

Furthermore, although mHealth apps may positively influence the user’s lifestyle on a short-term basis, the attrition rates are commonly high in long-term condition management [7]. Apps must be optimized to increase the adherence to the app-guided healthy lifestyles in order to practically reduce the user’s CVD risk factors. For example, avoidance of manual data entry increases app usage [8]. Providing immediate rewards in the form of incentives may be effective to motivate individuals to enhance physical activity [9]. A game-based design or positive peer influence may also be effective to increase user engagement, as is well shown in the Pokémon Go app [10]. To date, only few mHealth apps could demonstrate the positive impact of the user’s objective health status as represented by CVD biomarkers, such as blood pressure (BP), lipid profile, and glucose metabolism.

‘kencom’ is an mHealth app with integrated functions that include providing tailored health information, automatically gathered annual health check-up results, self-monitoring, and feedback service to the users. It is also synchronized to smartphone pedometers to count daily steps. The app is optimized to keep a low attrition rate through the easy-to-use user interface, incentive system, and regularly held events. Most importantly, the app data can be linked to a large-scale Japanese insurance database with detailed prescription information and to Japanese annual health check-up data, thus making a large longitudinal data set. In this study, we aimed to assess the effects of kencom on physical activity levels and CVD risk factors such as obesity, hypertension, dyslipidemia, and diabetes mellitus in a large Japanese population. We then discussed the plausible mechanisms of kencom from the perspectives of behavioral economics.

## Methods

### Overview and Functions of Kencom

**Figure 1:**
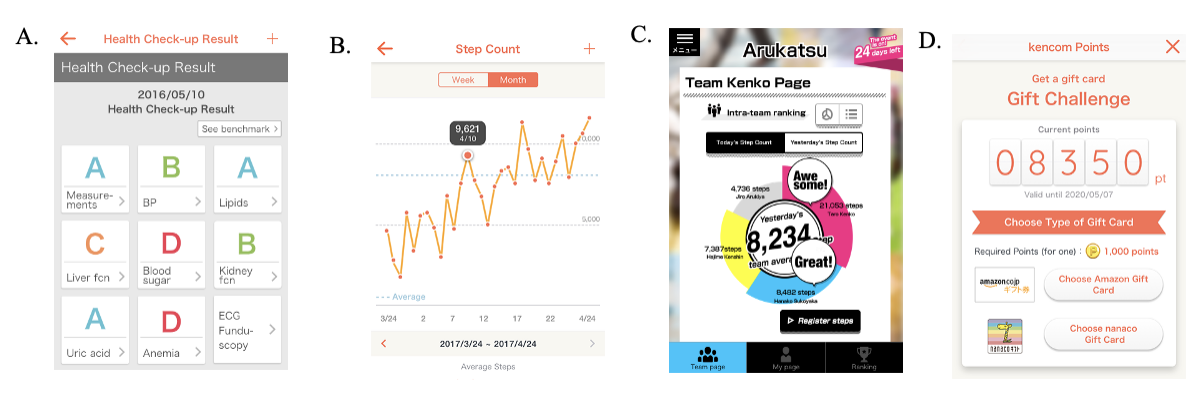
Sample images of kencom app. A. Annual health check-up results; B. Trends of daily step counts; C. Arukatsu (a game event) team page where individual step counts are shared with the team members, encouraging each member’s physical activity; D. Incentive system (kencom point).

kencom is a service developed by DeSC Healthcare, Inc. that is available as an app on iOS and Android platforms as well as through a web service. The kencom app is free to use and over 90% of the active users (ie, those who logged into kencom at least one time a month) are app users. Individuals can use the app for free if they live in Japan, are at least 19 years of age, and have joined an affiliated society-managed, employment-based health insurance association, one of the major insurers of Japanese universal health coverage. The affiliated society-managed, employment-based health insurance associations pay a subscription fee to DeSC Healthcare Inc. There are 5 core functions of kencom (Textbox 1; also see Figure 1).

**Core functions of kencom.**

1. **Displaying annual health check-up data**
  Users can check their own longitudinal, detailed health data based on the government-led annual health check-ups without manual input (Figure 1A).
2. **Monitoring daily health data and goal setting**
  Steps are counted by each smartphone’s built-in pedometer and the data can be synchronized to the kencom app with the user’s agreement. Users may set the daily goals of physical activity and the kencom app gives feedback to them according to self-checked achievements. Users may also manually input weight, blood pressure, and blood sugar levels in the app (Figure 1B).
3. **Providing tailored health information**
  Users receive tailored health information according to their lifestyle or disease risks, which is aimed to improve their health literacy. Every original article is peer-reviewed by medical doctors or nutrition professionals.
4. **Facilitating physical activity through team-based events**
  kencom regularly runs “Arukatsu” events to facilitate users’ physical activity. Up to 10 users form a team and the teams compete with each other in the total step counts for 1 month. Individual step counts are shared with the team members, encouraging each member’s physical activity. Game design elements were utilized in the event (Figure 1C).
5. **Incentive system**
  kencom points are awarded for daily logging-in and engaging in various services including Arukatsu. Points were not basically rewarded for daily activities or achieving goals. Users can get gift vouchers from kencom points that can be spent in the market place (Figure 1D).

### Data

This cohort study was based on 3 data sources: Japanese health check-up database, Japanese health insurance claims database, and kencom database. These data were integrated, anonymized, and stored in the affiliated local society-managed, employment-based health insurance associations. DeSC Healthcare Inc. then combined data from different society-managed, employment-based health insurance associations, making it a large, longitudinal database for research purposes. Multimedia Appendix 1 summarizes the variables from each data source. In brief, the Japanese health check-up database consists of the results of questionnaires, physical examinations, measurement of biomarkers, and imaging examinations, which are conducted annually for the majority of adults living in Japan. Japanese health insurance claims database records monthly information about the patient demographics, diagnoses according to the International Classification of Diseases and Related Health Problems, 10th Revision (ICD-10), medical procedures, and medications. The kencom database is mainly about daily physical activity data and the app usage.

The data anonymization was conducted under the “opt-out agreement” between the users and the society-managed, employment-based health insurance associations, in which the users were notified of their data usage and they can propose the deletion of their data. The study complied with the International Society for Pharmacoepidemiology Guidelines for Good Pharmacoepidemiology Practices. This study design was approved by the institutional review board of Juntendo University (approval number 2020045).

### Study Population

**Figure 2:**
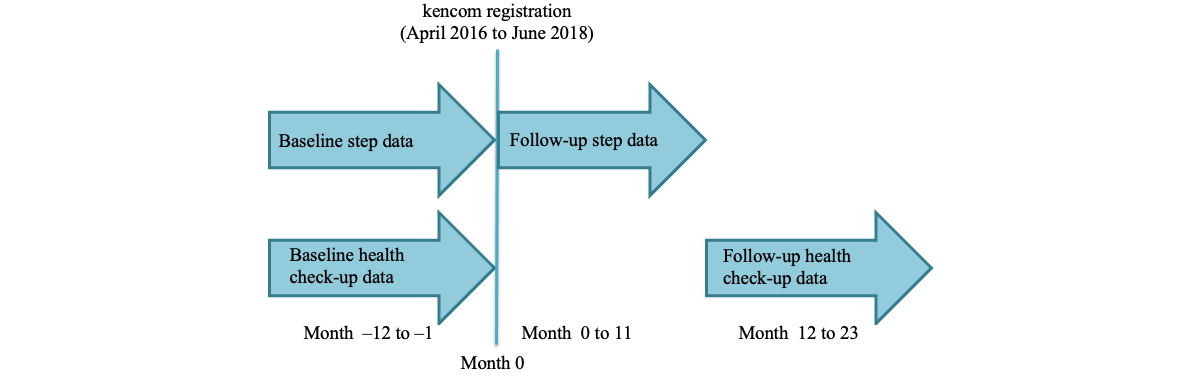
Data collection timelines.

**Figure 3:**
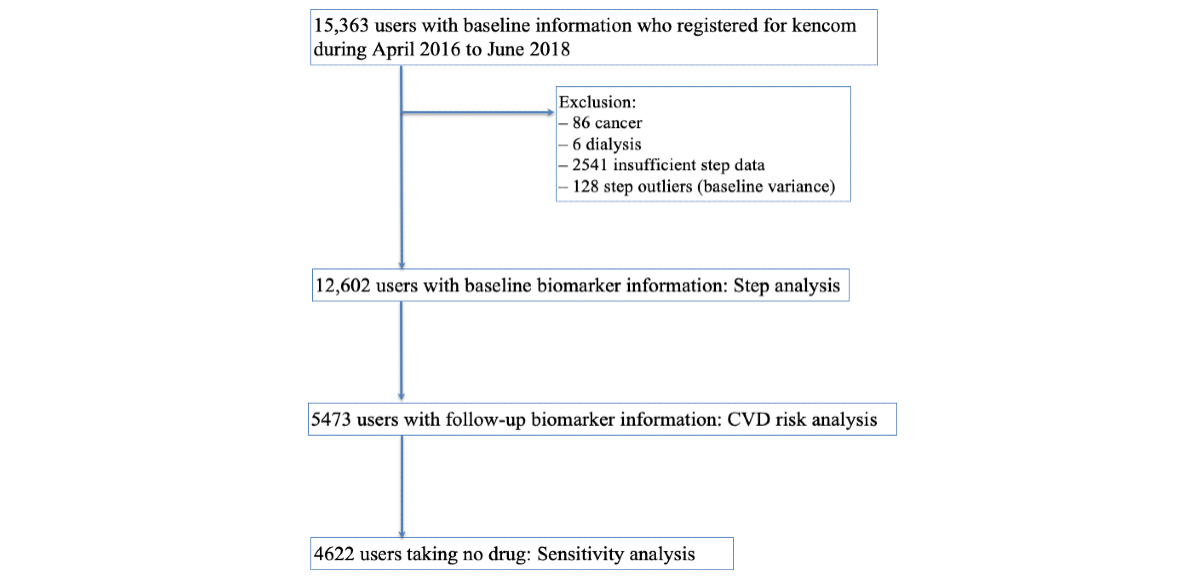
Study flow. CVD: cardiovascular disease.

This analysis targeted previously healthy adults living in Japan. The inclusion criteria were the registration of kencom between April 2016 and June 2018, and the availability of the baseline insurance claims data, health check-up data, and step data before and after kencom registration (12 months each) for the step analysis; and further availability of the follow-up annual health check-ups data for the CVD risk analysis. The baseline health check-up was defined as the check-up within 1 year before the kencom registration, and the follow-up corresponds to the first check-up conducted after 1-year usage of kencom (Figure 2). These criteria were applied only to iOS users in whom the step data prior to the app installation were automatically transferred into the app. The exclusion criteria included previous medical history of any cancer, end-stage renal disease requiring hemodialysis, and those with too high variance in baseline steps (top one percentile SD: over 7680 steps/day). From 15,363 kencom users with baseline information, 12,602 users met the criteria for the step analysis and 5473 for the CVD risk analysis (Figure 3).

### Step Analysis

Pre- and post-registration daily step counts were defined as the 1-year average step count before and after kencom registration (Figure 2). This definition allowed us to evaluate the change in step (post- minus pre-registration daily step count), minimizing the seasonal variation of step count [11]. The changes in average daily steps following kencom registration (ie, the mean of each user’s averaged steps over a year) were assessed for weekdays and the weekend. Furthermore, we compared the average daily step counts over each Arukatsu period, 1 month after the event, 1-2 months after the event, and 2-3 months after the event between the Arukatsu participants and nonparticipants.

### CVD Risk Analysis

The changes in CVD biomarkers (weight, systolic/diastolic BP, low-density lipoprotein [LDL]/high-density lipoprotein [HDL] cholesterol, triglyceride, and hemoglobin A_1c_ [HbA_1c_]) over at least 1-year usage of the app were assessed. Participants were divided according to the quintile of the changes in daily steps after kencom registration, and the changes in CVD biomarkers (follow-up minus baseline check-up data) were evaluated with the lowest quintile as the reference.

### Statistical Analysis

Depending on the normality of variables, continuous data were expressed as mean (SD) or median (IQR). The changes in daily step count or biomarker levels following kencom app registration were assessed by unpaired *t* test. We estimated the associations between the changes in CVD biomarkers and the changes in step count following kencom registration by multiple linear models with the biomarkers as the dependent variables. Multivariable model 1 was adjusted for age (continuous), sex (male/female), BMI (continuous), current smoking (yes/no), and alcohol drinking (yes/no) at baseline health check-up. Intention to improve lifestyle (“not interested,” “considering,” and “working on”) was further adjusted in multivariable model 2, because this question in the Japanese annual health check-ups has not been well scientifically validated. The linear trends in relation to the changes in CVD biomarkers’ levels were assessed with the use of quintiles of changes in daily steps following kencom registration as continuous variables in which the median change in the corresponding quintile groups was assigned. Sensitivity analysis was performed among 4622 users not taking any medications for hypertension, dyslipidemia, and diabetes mellitus. All analyses were performed using R 3.6.1 (The R Foundation). Statistical significance was set at a two-tailed *P*<.05 and multiple comparisons were adjusted by the Bonferroni method.

## Results

### Baseline Characteristics

Baseline cohort characteristics for the step and CVD risk analyses are summarized in Table 1. Among 12,602 users for the step analysis, the mean age was 44.1 (SD 10.2), and 52.25% (6584/12,602) were male. Distribution of age is illustrated in Multimedia Appendix 2. For the intention to improve their lifestyles, 22.9% (1225/5339), 44.6% (2380/5339), and 32.5% (1734/5339) of the users answered “not interested,” “considering,” and “working on,” respectively. The median (IQR) levels of BMI, systolic BP, LDL cholesterol, HDL cholesterol, triglyceride, and HbA_1c_ at baseline were 22.4 (20.4-24.7) kg/m^2^, 117 (107-128) mmHg, 119 (100-142) mg/dL, 63 (53-75) mg/dL, 80 (57-119) mg/dL, and 5.4 (5.2-5.6) %, respectively. The users accessed the kencom app for a median of 6.8 (IQR 2.6-14.1) days per month. The characteristics were similar to those of the population for the CVD risk analysis except for the male proportion (63.1% [3451/5473] in the CVD risk analysis).

**Table 1:** Cohort characteristics.

Characteristics			Step analysis (n=12,602)		CVD^a^ risk analysis (n=5473)
Age, year, mean (SD)			44.1 (10.2)		43.8 (10.2)
Male, n (%)			6584 (52.2)		3451 (63.1)
BMI (kg/m^2^), median (IQR)			22.4 (20.4-24.7)		22.5 (20.5-24.7)
Current smoking, n/N (%)			2724/12,389 (22.0)		1060/5463 (19.4)
Alcohol drink, n/N (%)			6408/8673 (73.9)		3473/4589 (75.7)
**Intention to improve the lifestyle, n/N (%)**					
	Not interested	1225/5339 (22.9)		685/2895 (23.7)	
	Considering	2380/5339 (44.6)		1258/2895 (43.5)	
	Working on	1734/5339 (32.5)		952/2895 (32.9)	
Systolic BP^b^ (mm/Hg), median (IQR)			117 (107-128)		117 (108-127)
Diastolic BP (mm/Hg), median (IQR)			72 (65-81)		72 (65-81)
LDL^c^ cholesterol (mg/dL), median (IQR)			119 (100-142)		119 (100-140)
HDL^d^ cholesterol (mg/dL), median (IQR)			63 (53-75)		61 (52-73)
Triglyceride (mg/dL), median (IQR)			80 (57-119)		82 (58-121)
HbA_1c_^e^ (%), median (IQR)			5.4 (5.2-5.6)		5.4 (5.2-5.6)
Average frequency of access per month, median (IQR)			6.8 (2.6-14.1)		6 (1.8-13.2)

^a^CVD: cardiovascular disease.

^b^BP: blood pressure.

^c^LDL: low-density lipoprotein.

^d^HDL: high-density lipoprotein.

^e^HbA_1c_: hemoglobin A_1c_

### Effects of Kencom on Step Count

**Figure 4:**
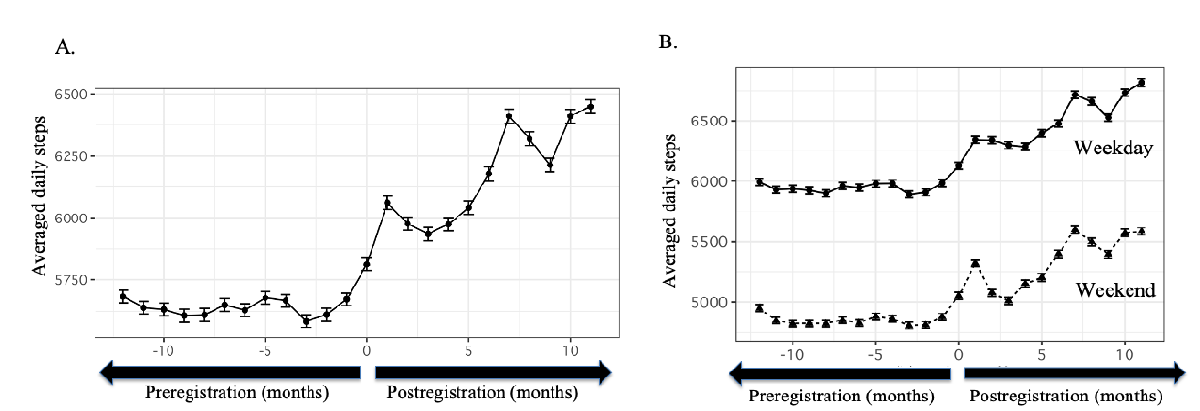
Changes in average daily step count following kencom registration. Dots indicate the mean daily step count of 12,602 users according to the months before/after kencom registration, and bars indicate the standard errors. In total cohort, the 1-year mean daily step count was increased from 5,642 steps/day to 6,152 steps/day following the registration (A). While the average step count was higher on weekdays compared with weekends, the increase in step counts was documented similarly for weekdays and weekends (B).

Mean step counts before and after kencom registration were 5642 (SD 2686) steps/day and 6152 (SD 2723) steps/day, respectively. The daily step count was significantly increased following kencom registration by a mean of 510 steps/day (*P*<.001). Figure 4A visualizes the change in averaged daily step count in each month before and after kencom registration. The increase in step counts persisted for a year after kencom registration. Physical activity levels were generally higher on weekdays than on the weekend, and the kencom app positively influenced both step counts (step change: 534 steps/day, *P*<.001 for weekdays; 463 steps/day, *P*<.001 for the weekends; Figure 4B). The spikes in month 1 and month 7 might reflect the Arukatsu event, in which users form teams and each team competes in step counts over a month, thereby exerting a positive peer influence on the participants to improve their physical activity levels. The company promoted kencom when the event was held, which led to increases in the number of users, highlighting the first spike at month 1. The event has been held almost 6 months apart; therefore, the users who experienced Arukatsu at month 1 were likely to experience the next event at month 7, represented as the next spike.

Table 2 describes the average daily step counts during and after each Arukatsu event comparing participants and nonparticipants. On average, the step counts were 1000-2000 steps higher in Arukatsu participants than in the nonparticipants during the event period. The differences in steps became smaller after the event, while generally the participants had higher daily steps than nonparticipants 3 months after the event.

**Table 2:** Association of Arukatsu event participation and average daily steps.

Event		N	During the event, mean (SD)	Event to month 1^a^, mean (SD)	Month 1 to month 2^a^, mean (SD)	Month 2 to month 3^a^, mean (SD)
**2016 event**						
	Participants	161	8103 (3943)	7067 (3020)	6693 (2807)	7105 (3019)
	Nonparticipants	2032	6575 (2553)	6433 (2576)	6160 (2534)	6549 (2677)
**2017 First event**						
	Participants	2017	8056 (3639)	7051 (2772)	6815 (2713)	6761 (2740)
	Nonparticipants	3374	6296 (2764)	6385 (2792)	6133 (2716)	5926 (2639)
**2017 Second event**						
	Participants	3083	7607 (4332)	6727 (3277)	6410 (3136)	6353 (2962)
	Nonparticipants	5306	6321 (2989)	6238 (2781)	5972 (2662)	6170 (2941)
**2018 First event**						
	Participants	6898	7030 (3890)	6274 (3289)	6063 (3305)	5966 (3109)
	Nonparticipants	5434	6317 (2786)	6415 (3129)	6190 (2921)	6046 (2915)
**2018 Second event**						
	Participants	5308	8295 (4205)	7242 (3471)	6865 (3409)	7203 (3595)
	Nonparticipants	6610	6327 (3156)	6095 (3040)	5876 (2998)	6193 (3149)

^a^Month X refers to the X month after the corresponding Arukatsu event.

### CVD Risk Analysis

Baseline/follow-up CVD biomarkers were available for 5473 users. They were classified according to the quintile of the change in daily steps (<–578, <–122, <248, <864, and ≥864) and the changes in CVD biomarkers following kencom registration were assessed. Baseline characteristics were similar across the quintile groups except for “intention to improve the lifestyle” (Multimedia Appendix 3). Higher increase in daily step count was associated with a higher prevalence of users already working on improving their lifestyles and higher average frequency of access to kencom.

**Figure 5:**
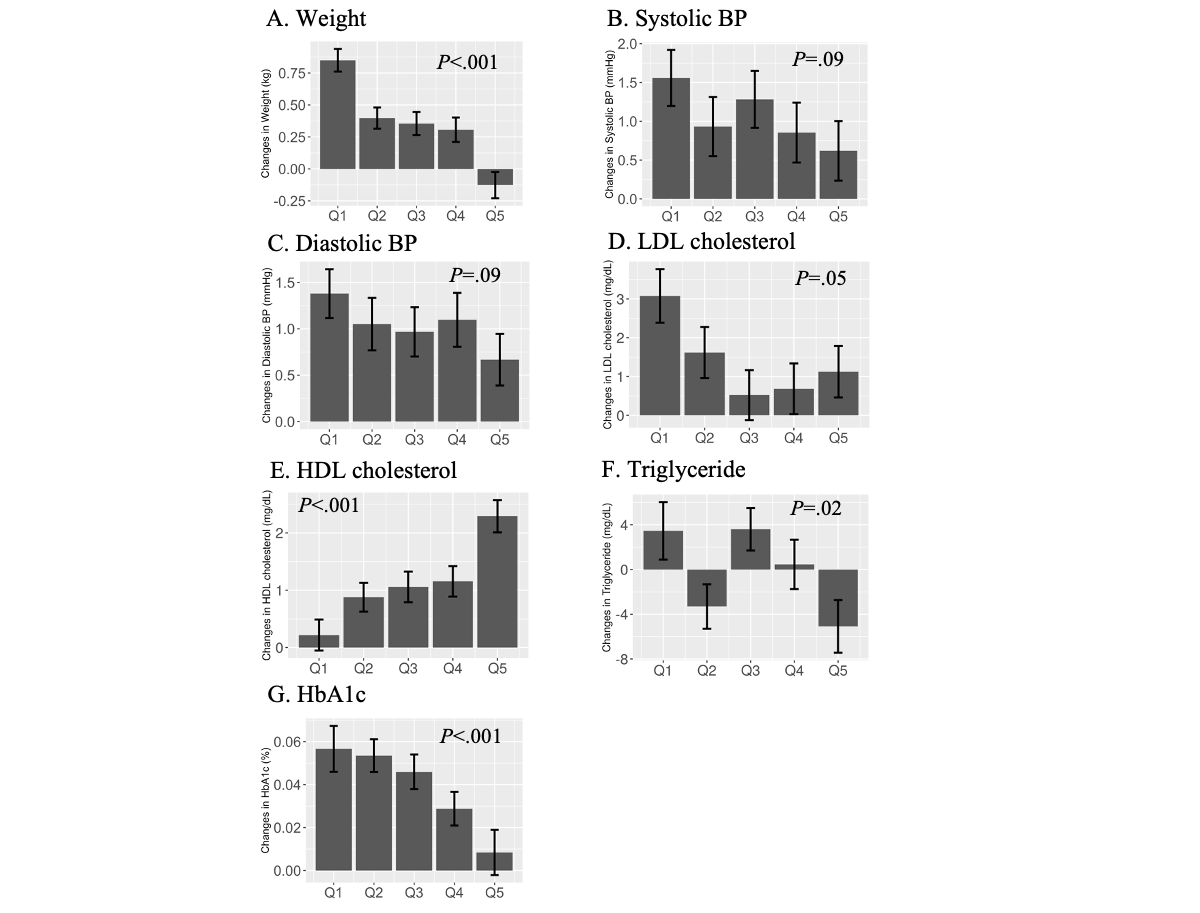
Changes in CVD biomarkers among users according to the changes in daily step count. Changes in CVD biomarkers following kencom registration (follow-up data minus baseline data) were compared according to the quintile of the changes in the user’s step count (corresponding to Q1 to Q5 in the x-axis). Bars represent the average changes in the biomarker in each unit and the error bars indicate the standard errors. *P* values are for the linear trend. BP: blood pressure; CVD: cardiovascular disease; HbA_1c_: hemoglobin A_1c_; HDL: high-density lipoprotein; LDL: low-density lipoprotein.

Increase in step counts was significantly associated with the improvement in weight, HDL cholesterol, and HbA_1c_ (Figure 5; *P*<.001 for all 3; the adjusted threshold for the *P* value was .007). In adjusted linear regression models, compared with the lowest quintile, the highest quintile in daily step change was associated with a significant reduction in weight (–0.92 kg, *P*<.001), LDL cholesterol (–2.78 mg/dL, *P*=.004), HbA_1c_ (–0.04%, *P*=.004), and increase in HDL cholesterol (+1.91 mg/dL, *P*<.001; Table 3). Further consideration of “intention to improve lifestyle” attenuated the improvement in LDL cholesterol and HbA_1c_ (*P*=.10 and .49, respectively), but changes in weight and HDL cholesterol remained significant (*P*<.001 each, respectively). The adjusted threshold for the *P* value was .007.

**Table 3:** Association between the changes in step count and the changes in cardiovascular disease risk biomarkers among 5473 users whose biomarker information was available^a,b^.

Cardiovascular disease risk biomarkers			Quintile 1		Quintile 2		Quintile 3		Quintile 4		Quintile 5		*P* value^c^
**Weight (kg)**													
	Model 1^d^	—		–0.43		–0.45		–0.53		–0.92		<.001	
	Model 2^e^	—		–0.39		–0.32		–0.50		–0.84		<.001	
**Systolic blood pressure (mmHg)**													
	Model 1	—		–0.66		–0.14		–0.35		–0.83		.22	
	Model 2	—		–0.97		–0.62		0.01		–1.01		.40	
**Diastolic blood pressure (mmHg)**													
	Model 1	—		–0.36		–0.08		0.24		–0.73		.21	
	Model 2	—		–0.26		–0.34		0.50		–0.51		.66	
**Low-density lipoprotein cholesterol (mg/dL)**													
	Model 1	—		–1.33		–2.85		–2.48		–2.78		.004	
	Model 2	—		–0.37		–1.92		–1.55		–1.87		.10	
**High-density lipoprotein cholesterol (mg/dL)**													
	Model 1	—		0.48		0.35		0.90		1.91		<.001	
	Model 2	—		0.47		0.38		0.73		1.97		<.001	
**Triglyceride (mg/dL)**													
	Model 1	—		–6.37		1.74		–3.95		–8.14		.04	
	Model 2	—		–4.82		7.80		–1.35		–8.10		.17	
**HbA_1c_ (%)**													
	Model 1	—		0.00		0.00		–0.01		–0.04		.004	
	Model 2	—		–0.01		–0.01		–0.01		–0.01		.49	

^a^A total of 5473 users are divided into 5 groups according to the quintile of the change in the daily step count following kencom registration.

^b^Values are the mean changes compared with quintile 1.

^c^Values are for the linear trend.

^d^Model 1 was adjusted for age, sex, BMI, smoking, and alcohol drinking.

^e^Model 2 was further adjusted for the intention to improve lifestyle (“not interested,” “considering,” and “working on”).

### Sensitivity Analysis

Sensitivity analyses were performed among 4622 healthy participants who did not take any medications for hypertension, dyslipidemia, and diabetes mellitus. They were divided into 5 groups according to their changes in step counts following kencom registration (Table 4). After full adjustment for the confounders, the highest quintile was associated with a significant reduction in weight (–0.75 kg, *P*<.001) and increase in HDL cholesterol (+1.84 mg/dL, *P*=.002) compared with the lowest quintile.

**Table 4:** Association between the changes in step count and the changes in cardiovascular disease risk biomarkers among 4622 users not taking any drugs for hypertension, dyslipidemia, and diabetes mellitus^a,b^

Cardiovascular disease risk biomarkers		Quintile 1	Quintile 2	Quintile 3	Quintile 4	Quintile 5	*P* value^c^
**Weight (kg)**							
	Model 1^d^	—	–0.45	–0.38	–0.49	–0.79	<.001
	Model 2^e^	—	–0.45	–0.26	–0.46	–0.75	<.001
**Systolic blood pressure (mmHg)**							
	Model 1	—	–0.49	–0.29	–0.20	–0.79	.26
	Model 2	—	–1.23	–1.06	0.01	–0.98	.51
**Diastolic blood pressure (mmHg)**							
	Model 1	—	–0.25	–0.22	0.02	–0.82	.10
	Model 2	—	–0.46	–0.67	0.12	–0.64	.48
**Low-density lipoprotein cholesterol (mg/dL)**							
	Model 1	—	–0.98	–2.53	–2.05	–2.72	.005
	Model 2	—	–0.10	–1.55	–1.29	–2.25	.06
**High-density lipoprotein cholesterol (mg/dL)**							
	Model 1	—	0.48	0.42	0.87	1.66	<.001
	Model 2	—	0.51	0.40	0.60	1.84	.002
**Triglyceride (mg/dL)**							
	Model 1	—	–7.44	0.07	–4.78	–7.11	.10
	Model 2	—	–6.43	6.97	–2.82	–6.64	.30
**HbA_1c_^f^ (%)**							
	Model 1	—	–0.01	0.00	–0.02	–0.03	.008
	Model 2	—	–0.02	0.00	–0.02	–0.02	.26

^a^A total of 4622 users taking no drugs for hypertension, dyslipidemia, and diabetes mellitus are divided into 5 groups according to the quintile of the change in the daily step count following kencom registration.

^b^Values are the mean changes compared with quintile 1.

^c^Values are for the linear trend.

^d^Model 1 was adjusted for age, sex, BMI, smoking, and alcohol drinking.

^e^Model 2 was further adjusted for the intention to improve lifestyle (“not interested,” “considering,” and “working on”).

^f^HbA_1c_: hemoglobin A_1c_

## Discussion

### Principal Findings

This is the first study to evaluate the effects of kencom, an mHealth app with integrated functions for healthy lifestyles, on physical activity levels and CVD biomarkers. The use of the kencom app was associated with increased daily step counts by a mean of 510 steps/day, and the higher increase in step counts was related to subsequent weight loss and improvement of lipid profile, in particular HDL cholesterol. The participations in Arukatsu events consistently had increased daily steps. Therefore, commitment to the kencom app might be associated with improved CVD risks through increased physical activity. Notably, this cohort study was conducted within the framework of kencom, in which the Japanese health data from multiple data sources were integrated; it may potentially become a much larger platform of anonymized health data where various epidemiological studies could be implemented.

### Association of Kencom and Steps

The present result of an averaged increase of 510 steps/day was not outstanding among incentive-based interventions, as a recent meta-analysis of 12 randomized controlled trials (RCTs) demonstrated an average increase of 607 steps/day by such interventions [11]. However, the observed changes in the activity level were after just registration of the app, unlike after the interventions of the RCTs. Furthermore, the meta-analysis had high heterogeneity due to the constituent small-sized RCTs and was also likely to be confounded by publication bias [11]. The present association of kencom registration and increase in daily step counts was robust because the analysis based on almost all-comers was the comparison of the average step counts over a year, separately in weekdays and weekends, thus accounting for the selection bias and well-established confounding by holiday period [4,12] and by seasons [13]. Although mHealth apps have typically high attrition rate that attenuates the effects [14], we observed that the increase in daily step counts after the registration of the kencom app was sustained at least one year, supporting the long-term efficacy of this app.

### Insight of Behavioral Economics Into the Mechanisms

Theories of behavioral economics may offer the mechanistic explanation as to why the kencom app can positively influence the user’s behavior [15]. The dual process theory describes 2 different systems that trigger human behavior: intuition or emotion-linked unconscious reasoning (System 1) and rule-based, analytic conscious reasoning (System 2) [16]. System 1 is susceptible to cognitive biases that can lead to “irrationality” dictated by the subjective reality, often precluding people’s health-oriented behaviors. The kencom app has been designed to modify the behaviors of individuals in contemplation stage in which status quo bias (preference for the current state) [17], present bias (tendency toward a small present reward compared with a big future reward), and overoptimism (unrealistic belief that one will less likely encounter negative events) play major roles in their decision makings. The app leverages functions that can work on System 1 and preclude the biases toward sedentary behaviors while accelerating positive biases such as peer bias. This characteristic satisfies the NUDGES framework as summarized in Table 5 [18].

**Table 5:** NUDGES framework of kencom.^a,b^

Framework components	Explanation	Corresponding features of kencom (examples)
i**N**centives	Incentives encourage the action	kencom points for daily logging-in and entry/winning of Arukatsu events; users can get gift vouchers from kencom points that can be spent in the market place
**U**nderstanding mappings	Understandings of the relations between choice and welfare prevent dropouts	Easy and detailed description of Arukatsu events to prevent dropouts; tailored articles and visualization of physical activity may appeal to the users’ emotion that healthy behaviors may lead to mental, physical, and financial benefit
**D**efaults	Better default settings improve the usability	Positive, big images like savory dishes or beautiful landscapes are displayed in the initial screen after logging-in (priming effect); no need to input step or health data by users
**G**iving feedbacks	Positive feedback keep one’s motivation	Setting of the daily goals of physical activity (declaration of commitment) and positive feedback according to the self-checked achievements; comment function in Arukatsu to encourage the team members with each other
**E**xpecting errors	Prevention of expecting errors enhances one’s commitment	One-click entry for Arukatsu to avoid procrastination; synchronization with smartphone accelerometers to keep daily record and minimize measurement errors
**S**tructure complex choices	Avoidance of complex options and optimization of the structures lead to less stressful experiences	The simple design allows anyone including those not familiar with a smartphone to use the app immediately; optimized user experience according to the frequency of user’s decision

^a^The features of kencom that satisfy NUDGES framework are summarized [18].

^b^The adherence to the NUDGES framework may lead to a better chance of altering one’s behaviors.

Some nudges have only short-term effects that inevitably limit the health benefits. The structure of kencom aimed to overcome this limitation by (1) providing multiple interventions in appropriate timings and (2) offering health education that may work on System 2 simultaneously with the nudge interventions. The following points illustrate the detailed interventions, potential effects, and the mechanisms of kencom:

- Incentives are offered for each daily login to kencom, aiming at habituating the behavior.
- Positive, big images such as savory dishes or beautiful landscapes are displayed on the initial screen after login, consistent with priming effects. The user’s positive feeling activated by the stimulus may sustain during the exploration of the app.
- The tailored health information is designed to be read like a newsfeed during commute or lunch time, when the users are not yet exhausted in a day and maintain their self-control. The contents generally focus on the “feasible action (plan),” with the intention to shifting the user’s fixed mindset into a growth mindset.
- The tailored health information is evidence based, which may improve the user’s health literacy.
- Arukatsu events are announced through the app and workplace. The participation may be prompted by the user’s fostered growth mindset, incentives, and peer influence from the colleagues.
- Arukatsu events leverage the use of game design elements in nongame contexts (competition of total step counts of the teams) [19], sharing of the step counts with team members, and comment function. These features may facilitate the participants’ physical activity through positive peer influence.

### Comparison With Other mHealth Apps

Other interventions leveraging cognitive biases have been evaluated for their efficacies in improving physical activities, including the Shape Up Rhode Island state-wide campaign [20], the SHAPE-UP program at a Northeastern university [21], and more recently, the CARADA app [22]. The concept of an event in the CARADA app was similar to Arukatsu, while the evaluation was conducted in much fewer participants (n=175) by one-time intervention, without any analysis on the postintervention periods [22]. Arukatsu events have been repeatedly conducted almost by half a year, which could foster the users’ participation and commitment to the events and apps, eventually contributing to the consistently increasing trend of steps after kencom registration.

The structure of kencom systematically differs from that of popular, scientifically evaluated mHealth apps such as Sweatcoin [4] and Carrot Rewards [23,24], in which incentive systems are leveraged as the main mechanism to stimulate the users’ physical activity; in Sweatcoin, digital currency was provided according to how many steps the users take; and in Carrot Rewards, users can earn loyalty points by achieving daily step goals. The “direct” incentives of these apps may efficiently enhance daily activity levels as the observational studies demonstrated an increase of over 1000 steps/day by using these apps. However, caution is needed to interpret these findings; the study on Sweatcoin could have serious selection bias because only 5892 out of more than 30 million committed users were analyzed [4]; and that of Carrot Rewards defined baseline steps based on the steps taken by the users during the 2-week induction period, potentially sensitive to the daily or seasonal variation [23], compared with the 1-year baseline evaluation in our study. In comparison, our analyses robustly support the association of kencom usage and enhanced physical activity with less bias, indicative of a novel mHealth approach toward population immobility without “direct” incentives.

### Implications on CVD Risks

The enhanced activity following kencom app registration was associated with improvement in CVD biomarkers, especially in weight and HDL cholesterol. This observation is in accordance with previous studies showing the positive relationship between walking exercise and HDL cholesterol [25,26]. Such an objective improvement in CVD risk factors further underscores the effectiveness of the use of the kencom app. Although the associations were consistent even after controlling for the semiquantitative intention to improve their lifestyles, the effect sizes were not clinically remarkable; this may highlight the characteristics of generally healthy, young participants and the variations in the users’ commitment on the app. Longitudinal analyses with longer follow-up data from multiple perspectives may confer the more precise and detailed effects and public health implications of the kencom app usage.

### Limitations

The strengths of our study are the large sample size, minimum exclusion criteria, rigorous definitions of baseline/follow-up step counts by averaging over 1-year data, and assessments of serial CVD biomarkers. Our study does have several important limitations. Study participants were mainly healthy Japanese and as such, our results may not be generalizable to other populations. Step data could be biased because the data solely from iPhone users were used, whereas there may be little difference in demographics between iPhone and Android users [27]. We cannot entirely rule out the possibility of unmeasured confounding factors, whereas the homogeneity of this study population minimized them. Finally, the current analysis was among users of the kencom app and a comparison with controls was not conducted. An RCT is warranted to infer causal effects of kencom app use on health outcomes.

### Conclusions

The use of an mHealth app “kencom” was associated with enhanced physical activity, which was related to the weight loss and increase of HDL cholesterol. This cohort study was conducted within the framework of kencom, in which health data from multiple data sources were anonymized and integrated, implicating the future usage to build a much larger database to provide reliable evidence.

## Appendix

Multimedia Appendix 1 Summary of the variables from each data source.

Multimedia Appendix 2 Age distribution.

Multimedia Appendix 3 Baseline characteristics across the quintiles of changes in daily steps following kencom registration.

## Abbreviations

BP: blood pressure
CVD: cardiovascular disease
HbA_1c_: hemoglobin A_1c_
HDL: high-density lipoprotein
LDL: low-density lipoprotein
mHealth: mobile health
RCT: randomized controlled trial
