# Supplementary material for: Effects of an mHealth App (Kencom) With Integrated Functions for Healthy Lifestyles on Physical Activity Levels and Cardiovascular Risk Biomarkers: Observational Study of 12,602 Users
Source: J Med Internet Res. 2021 Apr 26;23(4):e21622. doi: 10.2196/21622 (PMC8111509; doi:10.2196/21622)
Supplement: Multimedia Appendix 1 [file jmir_v23i4e21622_app1.docx]

Multimedia Appendix 1.Summary of the variables from each data source

| **Health-check data** | **Insurance data** | **kencom data** |
| --- | --- | --- |
| Date of medical checkup | Date of birth | Date of the steps, weight, BP etc. |
| Height | Gender | Steps |
| Weight | Date qualifications obtained/lost | Weight |
| Visceral fat area | Date of kencom registration | Blood sugar level |
| Waist circumference | Health insurance claim type | Systolic/diastolic BP |
| Systolic blood pressure | Treatment/prescription etc. start date | Arukatsu event details |
| Diastolic blood pressure | Treatment/prescription etc. end date (or duration) | Access details |
| Neutral fat | Outcome | Obtain/use of kencom points details |
| HDL cholesterol | ICD10 code | Daily goals details |
| LDL cholesterol | Cost | Achievement of daily goals details |
| HbA1c |  |  |
| Other biomarkers (e.g. GOT, GPT, creatinine, CBC) |  |  |
| Urine test (e.g. sugar, protein) |  |  |
| Use of medication for hypertension etc. |  |  |
| History of cardiovascular diseases etc. |  |  |
| Smoking |  |  |
| Regular physical activity |  |  |
| Eating habits details |  |  |
| Alcohol drinking, amount |  |  |
| Sleep quality |  |  |
| Intention to improve lifestyles |  |  |

The variables from each data sources were integrated, anonymized and used in the present analysis.
