# Supplementary figures and images for: Effects of an mHealth App (Kencom) With Integrated Functions for Healthy Lifestyles on Physical Activity Levels and Cardiovascular Risk Biomarkers: Observational Study of 12,602 Users
Source: J Med Internet Res. 2021 Apr 26;23(4):e21622. doi: 10.2196/21622 (PMC8111509; doi:10.2196/21622)

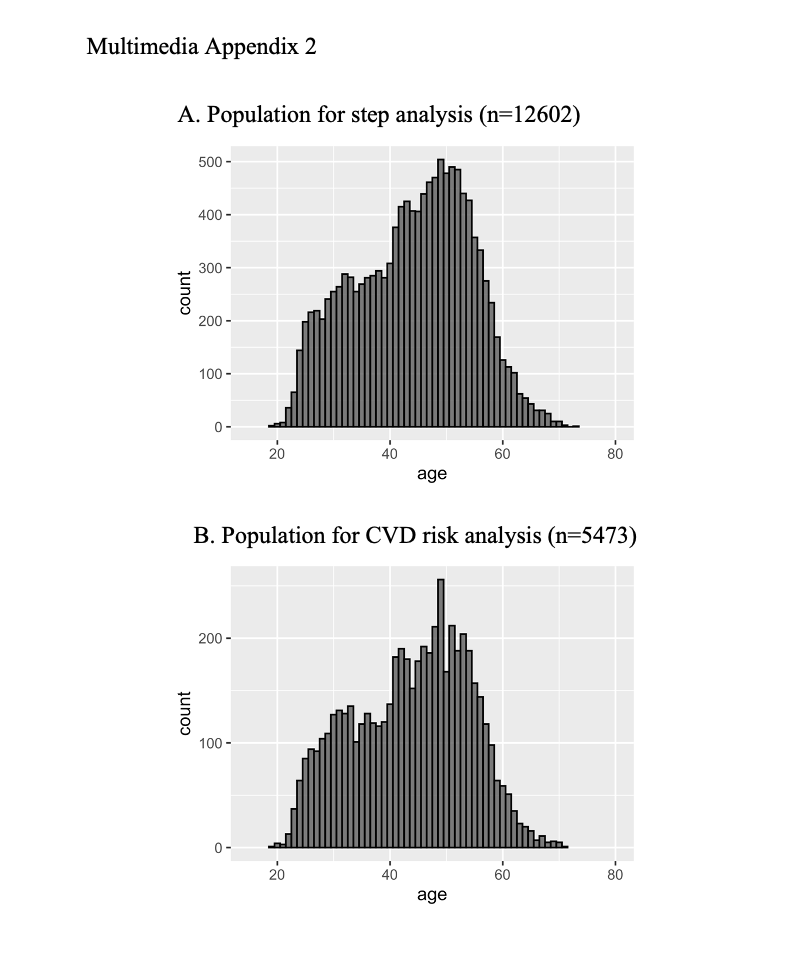

Supplement: Multimedia Appendix 2 [file jmir_v23i4e21622_app2.png]
