# Supplementary material for: Effects of an mHealth App (Kencom) With Integrated Functions for Healthy Lifestyles on Physical Activity Levels and Cardiovascular Risk Biomarkers: Observational Study of 12,602 Users
Source: J Med Internet Res. 2021 Apr 26;23(4):e21622. doi: 10.2196/21622 (PMC8111509; doi:10.2196/21622)
Supplement: Multimedia Appendix 3 [file jmir_v23i4e21622_app3.docx]

Multimedia Appendix 3. Baseline characteristics across the quintiles of changes in daily steps following kencom registration

|  | Quintile 1 | Quintile 2 | Quintile 3 | Quintile 4 | Quintile 5 |
| --- | --- | --- | --- | --- | --- |
| n | 1095 | 1094 | 1095 | 1094 | 1095 |
| Age, year | 42.4 (10.6) | 44.5 (10.0) | 44.5 (9.5) | 43.6 (10.1) | 43.8 (10.7) |
| Male | 822 (75.1) | 714 (65.3) | 637 (58.2) | 586 (53.6) | 692 (63.2) |
| BMI, m/kg^2^ | 22.5 [20.7, 24.8] | 22.6 [20.6, 24.6] | 22.6 [20.4, 24.7] | 22.3 [20.3, 24.7] | 22.6 [20.7, 24.7] |
| Current Smoking | 201 (18.4) | 208 (19.1) | 229 (20.9) | 208 (19.0) | 214 (19.6) |
| Alcohol drink | 786 (78.0) | 749 (76.4) | 678 (76.6) | 629 (72.0) | 631 (75.0) |
| Intention to improve the lifestyle |  |  |  |  |  |
| Not interested | 169 (25.3) | 178 (25.8) | 119 (20.3) | 109 (21.8) | 110 (24.4) |
| Considering | 287 (43.0) | 301 (43.7) | 268 (45.7) | 227 (45.3) | 175 (38.8) |
| Working on | 211 (31.6) | 210 (30.5) | 200 (34.1) | 165 (32.9) | 166 (36.8) |
| Systolic BP, mmHg | 117 [108, 127] | 117 [107, 127] | 118 [108, 126] | 116 [106, 127] | 119 [109, 128] |
| Diastolic BP, mmHg | 73 [65, 80] | 72 [66, 80] | 73 [66, 81] | 72 [65, 81] | 73 [66, 82] |
| LDL cholesterol, mg/dl | 117 [99, 139] | 120 [102, 141] | 118 [98, 139] | 119 [101, 142] | 118 [99, 141] |
| HDL cholesterol, mg/dl | 61 [52, 71] | 60 [51, 72] | 62 [52, 74] | 63 [52, 74] | 62 [52, 74] |
| Triglyceride, mg/dl | 82 [58, 122] | 85 [61, 124] | 83 [58, 120] | 82 [57, 121] | 78 [55, 116] |
| HbA1c, % | 5.4 [5.2, 5.6] | 5.4 [5.2, 5.6] | 5.4 [5.2, 5.6] | 5.4 [5.2, 5.6] | 5.4 [5.2, 5.6] |
| Average frequency of access per month | 4.4 [0.9, 10.8] | 4.2 [0.9, 11.4] | 5.3 [1.4, 12.1] | 6.3 [2.6, 13.9] | 9.5 [4.5, 17.1] |

Participants were grouped according to the quintile of changes in step count following kencom registration.

Values are mean ± SD, median (IQR), or number (%).

Abbreviations: BMI, body mass index; BP, blood pressure.
